# On the Treatment of Scarlatina

**Published:** 1879-05-20

**Authors:** T. R. Greenley

**Affiliations:** Kentucky


### Southern Medical Record.

EDITORS:

T. S. POWELL, M.D. W. T. GOLDSMITH, M.D. R. C. WORD, M.D.

*R. C. WORD, M.D., Managing Editor.*

☞All Communications and Letters on Business connected with the RECORD must be addressed to the Managing Editor.

VOL. IX. ATLANTA, GA., MAY 20, 1879. No. 5.

### ORIGINAL AND SELECTED ARTICLES.

### *ON THE TREATMENT OF SCARLATINA.*

BY T. R. GREENLEY, M.D., OF KENTUCKY.

It is a question whether scarlatina is a filth disease, or whether it may depend upon contagion for its epidemic character: for it must be admitted to be an epidemic disease, as it is common to most all countries. Most writers regard it as a contagious malady, and say that it depends upon contagion for its prevalence.

Formerly there was a distinction made between contagion and infection; but now the two terms are generally used synonymously. The word contagion is derived from the Latin contagio, to touch, and signifies the communication of a disease by contact. Infection, is derived from the Latin word inficio, and means the spreading of disease by exhalation from the lungs, or surface of the patient affected. These definitions and distinctions were made before the use of the microscope, and before it was known that infinitesimal particles of matter could float in the atmosphere. Confining our definitions to these Latin terms, we are still compelled to make some distinctions. There are certain diseases which depend on contact, or touch for communicability, as syphilis, psora, etc., but when we consider that particles of matter can escape from diseased bodies and penetrate the air, we are as much in contact with the disease, (the above named excepted) as though we had touched the patient: the only difference being we touch the product of the body instead of the body itself. In the venereal and some of the skin diseases it would seem that no particles of matter of a contagious or infectious nature escape so as to contaminate the air; hence it is essential that touch or contact should be practiced in order that the disease may be communicated.

Epidemic diseases may be contagious or not, but those that are contagious, are always due to contagion, and do not spread from any other cause; and if we find cases existing where there had been no possible chance of their being produced by exposure to the disease in others, we are bound to throw out of consideration the element of contagion as a cause.

Now I have attended many cases of scarlatina where the disease could not possibly be traced to contagion or infection. It is a very prevalent popular belief that scarlet fever is contagious, and consequently when the disease appears in a neighborhood, parents generally keep their children closely at home, and guard them against communicating with neighbors where the disease is prevailing; but as a general thing such precautions prove useless, as I have never known them to prevent the spreading of the disease. Then if we dismiss contagion as a cause of scarlet fever (which we must do if we know it to prevail independent of it, and as no disease can have two separate and distinct causes for its development,) we must consider it a filth disease and prevails from atmospheric influence. It is notable that dieases of this class, as erysipelas, diphtheria, etc., are of an asthenic character, and will not bear depletive or depressing treatment. You had almost as well cut a patient's throat, affected with either of these diseases, as to bleed him freely. Hence we should use supporting treatment, and not over medicate the patient.

A few years since there arose a controversy between some of the prominent members of the profession in Louisville, and the homeopathic physicians in regard to the success of the treatment of scarlatina. The homeopaths claimed that their plan of treatment was much more successful than ours. Whether they had a right to claim any such results I cannot say, but believe if their pretensions were based on facts, it depended more on withholding treatment, or medication, than on the virtue of their remedies. In other words, I concluded, perhaps *we* gave too much medicine.

In 1854-55 I practiced through an epidemic of scarlatina, and saw one hundred and twenty cases. I used very simple means in the treatment, mostly directing my attention to the kidneys and throat. The medicine consisted simply of spirits of turpentine, and sweet spirits of nitre, and stimulating and alterative applications to the throat when necessary. I had but few cases wherein sequila troubles supervened, such as dropsy, abscess, etc. Of all this number of cases *none* died. It may be said the disease was of a mild type, which was the case in perhaps twenty-five per cent., but a large number of them were very severe, requiring sometime to reduce the tumefactom about the throat so as to enable them to swallow the medicine. During the same period, in the hands of my neighbor physicians, twenty-one cases died. This result would indicate the advantage of my simple plan over the old mode of practice. I do not claim that this treatment would cure every case of scarlet fever, but I do think it is as successful as any I have yet heard of. The turpentine serves a double purpose; it not only acts as a diuretic, but also as an alterative application to the throat in swallowing. In cases of severe inflammation of the throat where there was a tendency to sloughing, I used very powerful applications, such as tincture of cayenne, iodine, etc., with volatile liniment exter nally.

In adopting the diuretic plan of medication I conceived the disease to depend on a blood dyscrasia, and as the skin was crippled and therefore could not perform its function, the kidneys could be safely taxed by way of compensation, in order to eliminate the blood poison from the system.

